# A Wearable Open-Source Neuroprosthesis/Neuro-Orthosis for Restoring Hand Function

**DOI:** 10.3390/s25113282

**Published:** 2025-05-23

**Authors:** Rune Thorsen, Maurizio Ferrarin

**Affiliations:** IRCCS Fondazione Don Carlo Gnocchi ONLUS, Via Capecelatro 66, 20148 Milano, Italy; rthorsen@dongnocchi.it

**Keywords:** myoelectrically controlled functional electrical stimulation, neuroprosthesis, stroke rehabilitation, spinal cord injury, open-source hardware, assistive technology

## Abstract

**Highlights:**

**What are the main findings?**

- An open-source, low-cost neuroprosthesis was developed to restore voluntary hand function in individuals with severe impairment.
- The system combines EMG-based intention detection with electrical stimulation in a wearable form factor, enabling hand opening and grasping.

**What is the implication of the main finding?**

- This work presents a complete, low-cost, replicable assistive device for the rehabilitation of functional hand use.
- The open-source design empowers researchers, clinicians, and users to adapt and further develop the system according to specific needs.

**Abstract:**

This paper presents a wearable, open-source system that combines electromyography (EMG) and functional electrical stimulation (FES) to restore hand function in individuals with disabilities caused by cervical spinal cord injuries or stroke. The device captures electrical signals produced during volitional muscle contractions and analyzes them to interpret the user’s intent to move. This information is then used to stimulate impaired muscles, promoting improved hand function and rehabilitation. We detail the design, prototyping, and testing of the system, emphasizing its modularity, affordability, and accessibility. Hardware and software, along with 3D-printable components, are shared via GitHub to enable replication and customization by professionals and makers. The system serves as both an orthotic device for enhancing grasping ability and a therapeutic tool for rehabilitating hemiparetic hands, with potential for broader applications. By addressing cost, customization, and accessibility barriers, this initiative promotes collaboration and further innovation in rehabilitation technologies, advancing the development of affordable, user-centered solutions for individuals with disabilities.

## 1. Introduction

Reduced hand function is a common consequence of conditions such as cervical spinal cord injury (CSCI), stroke, and other central nervous system lesions. CSCI often results in loss of grasp and manipulation abilities, due to paresis of the finger flexors, and people with tetraplegia prioritize regaining hand function [1].

Stroke, with much higher prevalence, frequently leads to paresis of finger extensors and spasticity of the finger flexors, where persistent muscle tightness causes the hand to remain clenched [2]. This inability to open the hand hinders the use of the affected limb, creates obstacles for task-oriented physiotherapy [3], and leaves few effective treatment options [4].

Functional electrical stimulation (FES) devices, such as the H200 [5], can activate paralyzed or paretic muscles, supporting movements as an orthotic (supportive) aid in neurological conditions [6]. However, enabling users to maintain direct control over the resulting movement remains a key challenge [2]. The integration of FES with voluntary muscle control has been shown to facilitate neuroplasticity, reactivating weakened synaptic connections between the motor cortex and spinal cord [7,8]. One effective approach is myoelectrically controlled FES, in which stimulation is triggered or directly controlled by the user’s own muscle activation. Electromyography (EMG) is used to detect residual voluntary muscle activity, even if minimal, and this signal is used to activate electrical stimulation of the corresponding or complementary muscles. This approach creates a closed-loop system that reinforces the user’s intention to move, thereby strengthening the brain–muscle connection. Triggered stimulation, which responds to voluntary movement, is particularly effective in improving motor function and reducing spasticity, reinforcing motor learning, and enhancing proprioception [9,10]. Furthermore, incorporating multiple feedback mechanisms, such as visual or sensory feedback, is essential to optimize rehabilitation outcomes by enhancing patient engagement and promoting active learning during the rehabilitation process.

Systems such as OmniHi5™ [11] offer some of these functionalities. This is the first commercial system to provide direct control of functional electrical stimulation (FES) using myoelectric signals (MESs). Such myoelectric-controlled FES (MeCFES), in which stimulation reflects the user’s voluntary muscle activation, enables precise timing and scaling of stimulation according to the user’s intent, promoting naturalistic, effort-dependent movement patterns.

However, a common limitation of commercially available systems is their lack of adaptability to individual functional, esthetic, and mechanical needs. Cost, limited customization, and design constraints often impact their suitability for individual use. Additionally, rigid certification requirements prevent modifications from meeting specific needs, and as closed systems, they are unsuitable for research aimed at improving them. Certification processes present a significant obstacle to introducing new technologies, inflating costs well beyond production costs. Furthermore, the financial risk for industry investment in such innovations is high if there are unresolved design issues.

As many assistive technology advancements tend to stall in the ‘valley of death’, a novel approach to development is proposed. Recent research has leveraged Free and Open-Source Software (FOSS) and Open-Source Hardware principles, as well as digital fabrication methods, to address these barriers by producing tailored, innovative devices that are affordable, functional, and tailored to individual users [12,13,14].

For individuals with cervical spinal cord injury (CSCI, tetraplegia), volitional wrist extension is often preserved and can be leveraged to control functional electrical stimulation (FES) of finger flexors. In a previous clinical trial, we demonstrated that a single-channel embodiment of myoelectrically controlled FES (MeCFES) functioned as a neuroprosthesis by enhancing the tenodesis grip, thereby supporting performance in daily activities [15].

In stroke rehabilitation, MeCFES can function as a neuro-orthosis by enabling volitional control of stimulation to support hand opening. Residual activity in wrist and finger flexors is used to modulate FES of the wrist and finger extensors, facilitating active extension. This approach has demonstrated the potential to reduce spasticity, enhance motor learning, and improve overall hand function. Our previous randomized controlled trial (RCT) confirmed the effectiveness of MeCFES in promoting hand opening and supporting functional recovery in individuals post-stroke [16].

Therefore, there is reason to believe that this approach has clinical relevance and should be made widely available as an effective method for rehabilitating hand function, both as an orthotic device and a therapeutic aid.

Open sourcing is the foundation of collaborative approaches, where making, modifying, and improving become community-driven tasks. This model has been successful in initiatives like Wikipedia and Linux, and is leveraged by commercial entities such as Google Inc. [17]. Although little has been investigated in the health sector, we hypothesize that open sourcing may challenge traditional models of technology transfer, making assistive technology more accessible and affordable. It may also foster awareness and upskilling opportunities and create spaces for individuals to understand and learn through this technology. It empowers people, especially within the disability community, to provide valuable feedback and support users throughout the process [18,19].

The wearable neuroprosthesis LibreMano One Aid (LM18) is presented here as a repository of descriptions and designs for healthcare professionals, makers, and, potentially, individuals with disabilities. It aims to provide an assistive solution, enhance skills, and foster empowerment. Due to its physiological effects, users of this material must read the disclaimers and exercise caution to avoid harm.

## 2. Materials and Methods

This system is a proof of concept intended solely for research and development purposes. The authors, developers, employers, and funding bodies disclaim all liability for its use. Users are fully responsible for ensuring compliance with applicable laws, regulations, and safety standards before testing or modifying this material. It is not approved for medical use and should be treated as a Class II device under relevant Medical Device Regulations. Use on living organisms requires prior authorization from appropriate regulatory authorities. Electrodes should be placed only on a single limb with firm skin contact, and the device must be powered off during handling or adjustments. It is unsuitable for individuals with pacemakers, implanted devices, or conditions sensitive to electrical stimulation, and should not be used during high-risk activities like driving. Only approved electrodes should be used, following all contraindications in the provided instructions. The symbol ⚠ (U + 26A0) must be clearly displayed on the device to warn users of potential risks. This disclaimer is not exhaustive, and users are advised to take additional precautions as necessary.

A fundamental principle is to enable voluntary movements to control stimulation proportionally, thus either copying or amplifying muscle contractions—a methodology that we have denoted by the acronym MeCFES.

Common adhesive ECG electrodes capture myoelectric signals (MESs) generated by muscle contractions. These signals are amplified and processed in real time to determine the appropriate intensity of electrical stimulation. Through digital signal processing, the system interprets the user’s voluntary contraction intention and adjusts the stimulation to provide precise, direct control over the resulting movement.

**Figure 1:**
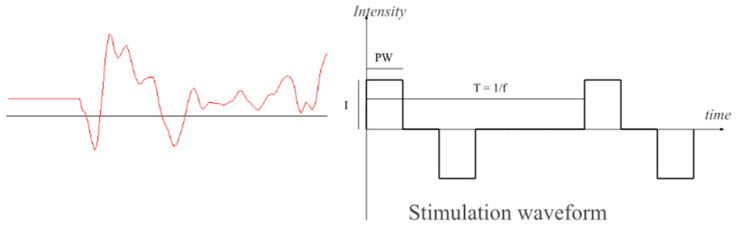
A 60 ms window of filtered MES (**left**) from a muscle stimulated with I = 15 mA. The stimulation (**right**) is a biphasic pulse doublet.

Stimulation is delivered transcutaneously using common adhesive TENS electrodes placed on the skin over the target muscle to deliver biphasic current pulses of PW = 300 µs duration at a T = 60 ms repetition rate (Figure 1). This doublet-form stimulation minimizes fatigue while generating tetanic contractions [20] and reducing interference with myoelectric signal (MES) amplifiers. These parameters have been validated in prior research as effective. The chosen frequency is a subharmonic of the power grid frequency; thus, both interferences can be suppressed with a simple comb filter. It also represents the lower end of the tetanic contraction threshold, minimizing fatigue induced by functional electrical stimulation (FES) and maximizing the interval between stimulation pulses, providing a window for sampling the volitional part of the MES. To prevent the stimulation pulses from contaminating the MES amplifiers and reduce this window, perfectly charge-balanced biphasic stimulation is critical. The integration of volitional control and FES results in a form of positive feedback, where small voluntary muscle activations are amplified through stimulation. While this mechanism underpins the intended servo-like control—where user intent produces proportionally larger movements—it also introduces the risk of instability. This is due to the potential contamination of the myoelectric signal (MES) by stimulation artifacts, which can be misinterpreted as volitional input, thus sustaining or escalating the stimulation unintentionally (latch-up). These artifacts can originate directly from stimulation pulses or indirectly from movement-induced disturbances at the electrode–skin interface, causing a shift in the half-cell potential of the electrodes (movement artifacts).

Previous work has addressed these challenges in detail. Reference [21] describes an amplifier design that suppresses such artifacts, while [22] presents a stimulator architecture that minimizes false-positive feedback and discusses strategies to improve system stability. These include the use of MES blanking intervals and dynamic adjustment of amplifier gain and offset, as elaborated in the later section on signal processing. If the loop gain—including artifact contributions—exceeds unity, the system may enter a latch-up state, leading to continuous stimulation regardless of user intent. As the comb-filter removes DC components, this may also manifest as a full on–off oscillation. Therefore, a watchdog mechanism must detect and terminate prolonged maximum stimulation, ensuring user safety and restoring control integrity.

Commercially available surface electrodes are a simple, non-invasive alternative to implanted electrodes, but (in addition to poor muscle specificity) they require donning and doffing for each use and they must be placed precisely over the target muscles to ensure effective stimulation. People with tetraplegia have been able to autonomously place electrodes during trials [15], so manual electrode placement is viable but not optimal. Therefore, an ‘electrode applicator’ method is needed to facilitate autonomous donning and doffing and some have proposed a splint-based solution [5]. Such an electrode applicator may also have a stabilizing effect, reducing the motion artifacts previously described. Surface electrodes rely on current passing through the skin, requiring a conductive interface, typically water-based (electrolyte). Investigations into dry electrodes, compared to standard ones, have also been explored.

**Figure 2:**
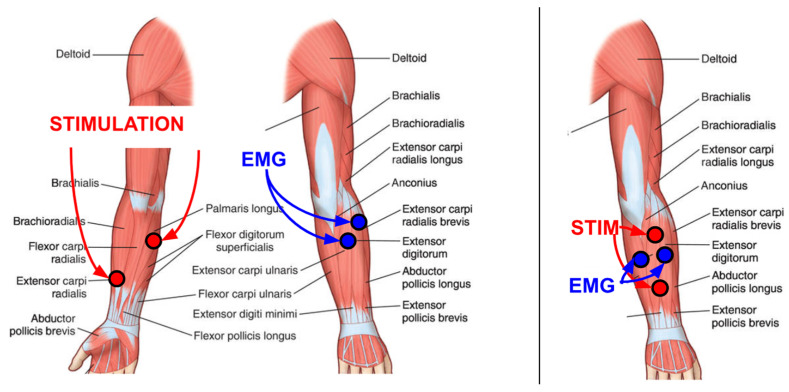
Indicative electrode positions for enhancing tenodesis grip (**left**) and training wrist extension post-stroke (**right**).

Depending on the rehabilitative objective, various electrode configurations can be used, but specifically for reinforcing the tenodesis grip and training hand opening, the electrodes can be placed as shown in Figure 2. Various therapeutic strategies may require more advanced techniques [16]; for example, linear combinations of two channels can allow wrist extension and anterior deltoid control to work in synergy with the opening of the hand for reaching. Only when the patient actively uses both muscles does the stimulation induce hand opening. A variant of this approach involves allowing the activity of the long flexors of the fingers to inhibit stimulation controlled by wrist extensors, promoting the unlearning of undesirable co-contraction of antagonist muscles.

## 3. Results

### 3.1. System Description

LM18 is a myoelectrically controlled functional electrical stimulation device with two inputs and two outputs. It is designed for two primary user scenarios: (1) assisting grasping (tenodesis grip) and (2) reeducating hand opening.

The project repositories are hosted on GitHub, see data availability, and we have also published a video showing how a person with tetraplegia can regain hand function, grasping and releasing objects and other activities of daily living, using a previous version of the device [16].

The circuit supports two sets of recording and two sets of stimulation electrodes (two-by-two channels), allowing for independent control of two muscle groups. This feature enables versatile use in various rehabilitation scenarios.

The systems repositories are structured as follows:

- LM_HW: contains the electronic circuit (schematics and PCB designs) for recording myoelectric signals (MESs), processing data (LM_FW firmware), delivering functional electrical stimulation (FES), and enabling wireless communication with the LM_APP.
- LM_FW: hosts the embedded firmware responsible for processing signals and controlling stimulation delivery.
- LM_APP: includes software applications with a graphical user interface (GUI) for operation on screen-based devices.
- ElAp: designs for 3D-printable devices placing electrodes on the arm.

The beta design of the LM_HW electronic circuits was validated through ten functional prototypes, assembled by an online manufacturer at a total cost of EUR 2000, averaging EUR 200 per device. This includes EUR 160 for the printed circuit board (PCB), EUR 520 for assembly, and EUR 1320 for components, covering materials and overhead.

**Figure 3:**
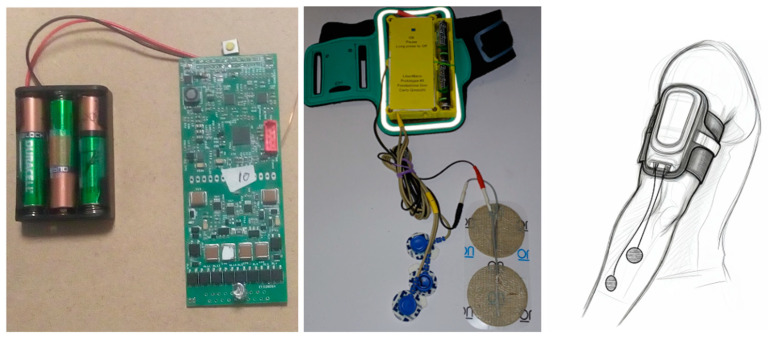
The assembled electronic PCB part and a battery pack. The centerline is where the circuit can be folded in two to increase compactness. It fits in a runner’s armband (for a 5-inch smartphone) placed on the upper arm.

The populated PCB, see Figure 3, measures 10 cm × 4 cm × 0.5 cm and operates on standard disposable, rechargeable, or lithium batteries. Using a 3D-printed case, it can be placed in a runner’s cellphone holder on the upper arm or directly within the ElAp applicator. Optionally, it can be folded along the midline for a more compact version.

### 3.2. LM_HW Hardware Description of the Circuit Board

**Figure 4:**
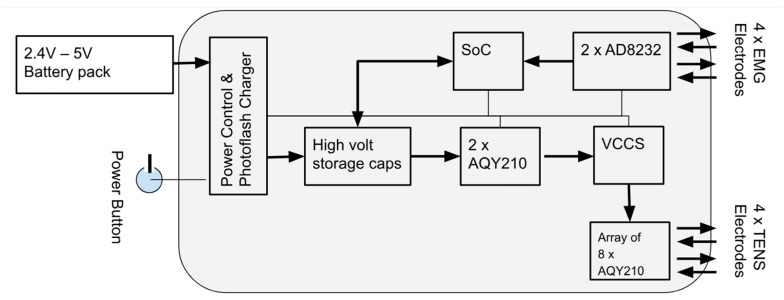
System block diagram showing power and electrode interface.

The electronic circuit integrates a modern system-on-chip (ADuCM360) with two myoelectric signal inputs and two stimulation outputs, see Figure 4. The input uses a biopotential amplifier (AD8232), a high-performance signal acquisition system chip with high common mode rejection. In particular, it features a fast recovery circuit, which enhances robustness against stimulation spillover and artifacts solving issues present in early systems using nonlinear feedback [21], making it suitable for muscle signal processing in environments with electrical stimulation.

The stimulation is generated by a single high-voltage voltage-controlled current source [22] using PhotoMOS (AQY210) solid-state relays for time multiplexing four individually controlled current pulses into two channels of biphasic stimulation. To generate the high voltage, up to 320 V, a photoflash capacitor charger IC (LT3484) with a compact transformer (LDT565630T) provides high efficiency (>85%).

The ADuCM360 interfaces with a graphic user interface host via a wireless (Wi-Fi/Bluetooth) module. A single push button is used to switch the device on and off.

The device has no display, but can be connected to a PC, smartphone, or tablet that runs the graphical user interface (GUI), which is used for setup or advanced uses of the device, but the device works in standalone mode.

### 3.3. Choice of Key Specifications

Power supply is a critical consideration for this application, as the device must always be ready for use. The chosen buck–boost converter, the TPS6102, offers a wide input voltage range of 2.4 V to 6.5 V, enabling the use of various battery types, including AA, AAA, or lithium batteries.

The LM18 prototypes have an average standby consumption of 12 mA, providing days of autonomy. However, the active consumption depends on the need for stimulation, and we estimate that a fully charged Li-ion battery (1400 mAh), fitting on the backside of the PCB, should provide at least one day of active use.

Some devices, like H200, regulate intensity in 1 mA steps, but the recruitment curve of muscles may be very steep, and some patients have reported the need for fine-grained regulation. Therefore, we have used a 12-bit DAC to control currents from 0 to 100 mA in 0.024 mA (24 μA) steps. However, a default (adjustable) software setting will limit the current to 30 mA, and the 320 V differential voltage supply from the LT3484 will also limit the effective current depending upon electrode impedance.

One of the important parameters for recording a clean MES is minimizing interference from the output-to-input leakage current. The LM18 is below the measurable threshold of standard lab equipment (<1 μA), with undetectable output-to-input charge injection (<10 nC). The choice of the PhotoMOS is dictated by the voltage and a timing much faster than the intra-pulse width t(on/off) < 300 μs.

### 3.4. Connectivity

Given that GUI programming typically demands significant design and implementation resources, the decision was made to delegate UI activity to external devices, allowing relevant signals to be relayed to screen-based devices running the LM_App software. The basic version uses a Bluetooth module (WT12) for wireless communication with the GUI host. Additionally, a Wi-Fi connection can be established using the low-cost ESP32 module, providing greater flexibility for various GUI host systems. A very flexible communication protocol has been developed. It is simple but powerful in that it lets the LM_App use the device as a random-access memory based upon the firmware variables, thus accessing any parameters and signals processed in the LM_FW.

### 3.5. LM_FW Firmware and Digital Signal Processing

The firmware handles the signal processing, converting the muscle signals into stimulation.

The ADuCM360 is flashed with the software (firmware) for real-time MES analysis, including offset adjustment, comb and RMS filtering, blanking, and dynamic control of stimulation levels, with signal processing parameters accessible by a dedicated communication protocol via a Wi-Fi/BT interface. The device can be powered off from the app or will auto power off when not used.

Volitional MES is estimated from the smoothened value of the RMS of the filtered signal of each channel, the volitional control signal estimate (*V*). After subtracting an offset representing involuntary background noise (*Off*), it is multiplied by a gain (*G*) factor to form a smooth control signal. The output (*I*) of each stimulation channel is the combination (sum or difference) of these control signals, permitting synergies or inhibition of the channels, see Equation (1).

*I1* = (*V1* − *Off1*)·*G1* + (*V2* − *Off2*)·*G2* (1)

### 3.6. Graphic User Interface Client

Once the system is calibrated for the user, it is self-contained but for calibration purposes, and for biofeedback purposes a graphic user interface software (GUI host) is published, with the first generation written in Android Studio as an Android Application downloadable as an APK from the repository. The second-generation GUI is written as a progressive web app. The latter is a device-antagonistic version that runs in any modern browser with Bluetooth. If Bluetooth is not available, the HW has to be enhanced with the ESP32 to provide direct Wi-Fi connection.

**Figure 5:**
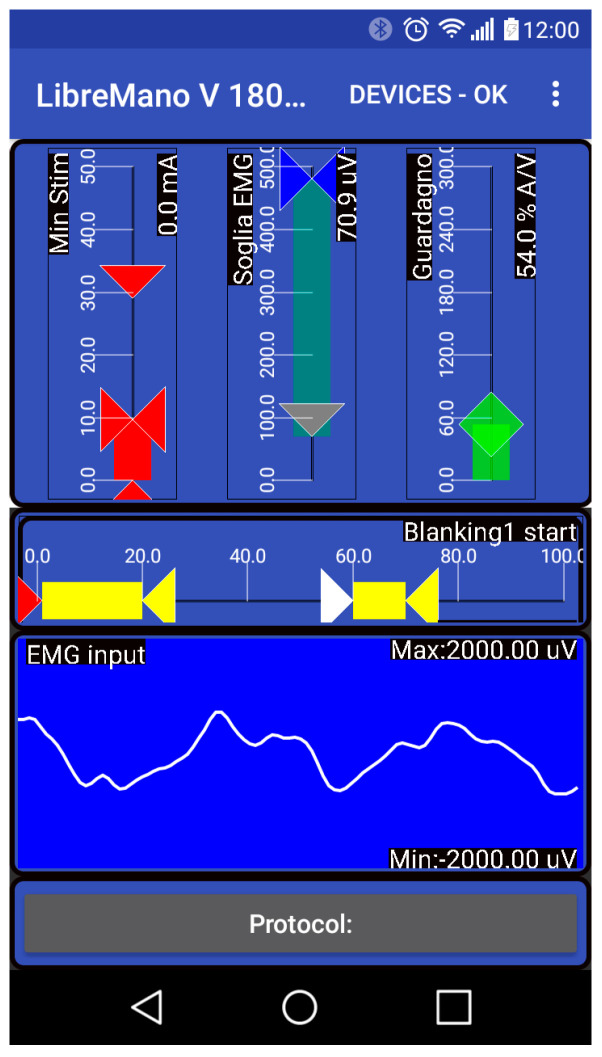
LM_App GUI. Screenshot of the graphical interface showing the current stimulation level (Red) in the middle; the current EMG level (blue); and to the right the sensitivity or gain (green). Inspection of the signal and other parameters are shown below.

The functional part of the GUI holds sliders and a signal display (Figure 5). Monitoring the signal is useful to check that electrodes are correctly located, control the quality of the recorded signal, detect faults like peeling electrodes, and set signal processing parameters. Furthermore, it is useful as biofeedback to the user. Vertical sliders set the range of stimulation, offset, and gains and how channels interact. Horizontal sliders provide an intuitive way to blank out periods of non-volitionally related stimulation responses (artifacts, m-waves, h-reflexes, and f-waves), see Figure 1, in which the first 20 ms of signal is zeroed. Similar screen panels show the other channels and how they can be combined, assigning positive or negative gains.

### 3.7. ElAp—Electrodes and Electrode Applicator

The first step in testing the method with any person is to use manual placement using adhesive electrodes, but for recurrent use we envisaged an electrode applicator (ElAp) design to host electrodes. Commercial electrodes use a kind of sticky hydrogel as a conductive medium to keep impedance low, but doffing the device became an issue due to the stickiness. Therefore, biocompatible dry electrodes [23] were tested and compared to standard EMG electrodes. However, it was found that signal quality deteriorated with dry electrodes, as stimulation artifacts increased with electrode impedance.

Chamois leather, cut to fit the size of the electrodes, proved useful as it adheres well to both adhesive electrode types, providing an easily repositionable, detachable, and remountable solution, maintaining conductive characteristics. A well-known characteristic of chamois leather is its ability to stay wet, making it comfortable on the skin during prolonged use. The electrodes can be easily wetted, and the chamois did not dry out during hours of application.

### 3.8. Anthropometric Analysis and Modeling

**Figure 6:**
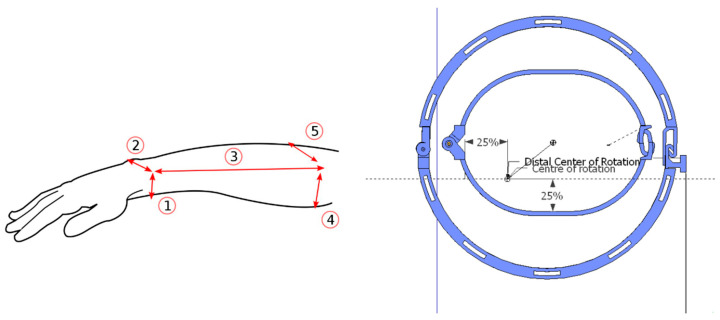
Measurements of the forearm: 1—wrist height; 2—wrist width; 3—forearm length; 4—forearm height (the wider part); and 5—forearm width (the wider part). To the right, a left-hand DP and PP.

Previous clinical trials and fieldwork have shown that electrode placement varies based on individual anatomy and neurological conditions, making standardization challenging, and we found that a common strategy, using tenodesis grip, is supination of the hand during grasping. This requires relative rotation between the distal and proximal forearm segments. A rigid model would constrain this motion and is a commonly heard argument against splint-based solutions. Therefore, the model must allow for independent rotation of the distal segment, aligning with the anatomical axes of rotation of the bones, radius, and ulna. A dynamic model incorporating these rotational degrees of freedom was constructed using mechanical simulations. A concept was developed to utilize the wrist as both an orientation point and a fixation anchor, extending its reach below the elbow as a two-part solution: a proximal part (PP) that attaches around the wrist, and a distal part (DP) connected with a structure that anchors electrodes along the forearm, see Figure 6.

Modeling biodynamics is a complex task that typically requires professional-grade software, while FOSS solutions for modeling dynamic movements are scarce. To address this gap, a modified version of SolveSpace has been developed and is available in an accompanying repository. Simulations showed that the rotation of the radius around the ulna involves the radius moving within the sigmoid cavity of the ulna above and around it below and a relevant observation was made. The distance between the distal part (DP) and the proximal part (PP) changes during forearm rotation (pronation/supination), varying by up to 5 mm.

To provide unconstricted movement, the connection between the DP and PP must be flexible or have joints and it was found that rigid connections must be aligned with the radius and ulna, moving parallel to the bones during rotation.

One of the design files incorporates a novel mechanism that allows translation along the forearm’s rotational axis with mechanical connectors linking the distal and proximal parts of the applicator, with a 3D-printable hinge solution selected for affordability and ease of production.

In a biomechanical model with components 180 mm apart, the bars must move 15–20° in their sockets to allow rotations to 45°, 90° (anatomical normal), or 110°, accommodating varying distances between parts. Anatomically, the rotation center of the proximal part is central, while that of the distal part is located one-quarter posterior and one-quarter external to the ulna’s center. A forearm length may reach 250 mm, which allows diagonal placement in a 3D printer with a 20–30 cm plate. The proximal circumference was found to be very dependent on fitness and build, but largely circular in the range of 200–300 mm. For the DP, a wrist circumference around 170 mm with wrist height and width around 40 mm and 60 mm was used. These values serve as starting (default) parameters for parametric designs, where the size can be adapted to the user by measuring the parameters and inputting them into the CAD drawing variables.

**Figure 7:**
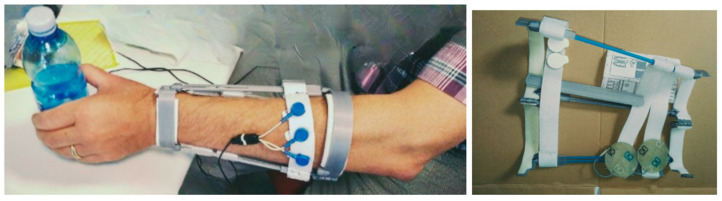
Functional testing with an ElAp using elastic Velcro straps to place the electrodes, allowing for rotational freedom of the wrist and hand.

The shown design requires 3D printing of 11 components, and assembly using Velcro straps and electrodes, as described (Figure 7). The DP and PP are two hinged half-circles that, through a four-hinged bar system, connect with each other. The user can hook their fingers (if paralyzed) around the inner bar to open the ‘cage’. Sets of elastic Velcro straps slide along the bars, providing support for the electrodes, which have Velcro backings for attachment. An experimental version of the ElAp can house the electronic device, forming a complete self-sufficient system. Printing the ElAp, with a mass well below 100 g of PLA, would take approximately 3–4 h. The material cost for the PLA, Velcro, and miscellaneous components is under EUR 10, though the initial cost of purchasing whole packs of consumables should be considered. Additionally, labor costs must be factored in. The costs of electrodes and chamois leather are recurrent and depend on factors such as country and quantity.

We also tested using an elastic garment for covering the ElAp, and placing electrodes was tested as well and found feasible. However, this adds significant complexity to the system, as sewing is a different technology and not quite accessible as a digital fabrication methodology yet.

## 4. Discussion

The potential for restoring movement through functional electrical stimulation (FES) has advanced since Vodovnik’s seminal studies [24], but translating these systems into clinically usable solutions remains challenging. High certification costs and the resulting lack of system flexibility hinder customization and limit their appeal to investors and end users [25,26]. We believe open-source approaches can bypass traditional barriers, making assistive devices more accessible and affordable.

We have presented a framework for a fully downloadable, ready-to-build neuroprosthesis, utilizing FOSS and digital manufacturing techniques, including outsourced PCB production, assembly, and 3D printing.

We have demonstrated that digital fabrication (online services for electronics prototyping, 3D printing, and free software development tools) enables flexibility in research and development while offering a cost-effective alternative to commercial solutions. Key cost factors include the use of state-of-the-art, specialized components, which are expensive, and the number of channels required. As with any manufacturing process, unit prices decrease with quantity. For example, a batch of 10 prototypes fabricated by a European online service for this project cost approximately EUR 200 per unit. If a 3D printer is available, the cost of materials and machine time is much lower, although this comes at the expense of man-hours. For hobbyists or makers, this time may be considered ‘free’, but for professionals, it can be a significant cost. This presents an interesting area for future research in participatory action research on assistive devices.

The electronic component of the project has been tested and developed in clinical trials, but further development in system robustness, signal processing, and, most importantly, design is needed to create an ergonomic, fit-for-purpose, and easily applicable device. Alternatively, the circuits can be placed in a simple 3D-printed case and carried in a runner’s armband on the upper arm. This setup offers flexibility to modify the electrode configuration as needed, facilitating hygiene (electrodes can be easily disposed of after use), but at the cost of additional time required for preparation and mounting.

While a comparative analysis would be valuable, we have not identified similar systems with sufficient disclosure of cost structures, technical documentation, or design flexibility to enable a meaningful comparison. This highlights a key advantage of our open-source approach: transparency in design, implementation, and cost, which supports reproducibility, customization, and informed decision-making for both researchers and clinicians.

R&D professionals and makers looking to further develop the system should familiarize themselves with relevant standards. Ensuring compliance from the outset facilitates smoother integration into clinical and real-world settings.

User feedback from clinical trials, such as the findings reported in Thorsen et al. (2020) [15], provides valuable insights for those entering the field. In our experience, some individuals, also neurologically normal, experienced significant discomfort, exhibiting hypersensitivity at very low stimulation levels (<5 mA), well before muscle activation was achieved. Therefore, starting with lower intensities and gradually ramping up to the desired level is a key practice when deploying such devices.

The chosen buck–boost converter, the TPS6102, provides a wide input voltage range, with the ability to operate from as low as 0.9 V. This makes it theoretically suitable for powering devices via emerging energy harvesting methods. The firmware (LM_FW) was developed to enable precise EMG signal acquisition and control of functional electrical stimulation (FES), while the graphical user interface (LM_APP) was concurrently created to facilitate device configuration and performance monitoring by therapists and researchers, as well as to serve as a biofeedback tool for the patient.

The interface design has evolved through iterative development cycles, informed by usability studies involving individuals with spinal cord injury and stroke [15,16]. While WCAG compliance was not a central goal in this version, the open-source nature of the project offers the potential for community-driven extensions, including accessibility improvements and custom skins. Future versions of the system will explicitly aim to align with WCAG accessibility standards to enhance inclusivity and usability.

However, the rapid evolution of Android platforms has made it labor-intensive to keep up with ever-changing safety requirements. The installable APK requires Android Marshmallow or older versions but benefits from not requiring Wi-Fi or internet access.

An alternative approach is the use of a progressive web application (PWA), which operates offline once loaded. It is installation-free as long as there is a modern browser, providing the added advantage of being operating system (OS)-agnostic. This means users do not need to install specific software, unlike traditional applications built in C++ or Java. Additionally, the PWA enables remote updates and troubleshooting, allowing therapists to connect with the device remotely via the user’s GUI host, potentially enabling telerehabilitation. The PWA is where further software development should be focused.

Continuous clinical monitoring and adjustments are crucial for optimizing assistive devices [27]. Rehabilitation devices should therefore not be considered pure DIY projects but should evolve into co-design efforts involving healthcare professionals, prioritizing safety and security. The importance of functional evaluation indicators in assessing the effectiveness of the neuroprosthetic system cannot be overstated. In previous clinical trials [15,16] as well as more recent works by the authors, functional outcome measures such as the Action Research Arm Test (ARAT) [28] and the Individual Prioritized Problem Assessment (IPPA) [29] have been employed to evaluate the impact of assistive technologies on patients’ functional abilities. These metrics provide a comprehensive view of upper limb recovery and patient-centered outcomes, respectively. Future trials utilizing the open-source system could benefit from incorporating these or similar measures [30] to objectively assess improvements in motor function and patient-reported outcomes. We recommend that future adopters of this system consider these established metrics to support evidence-based validation and further development of the technology.

## 5. Conclusions

This paper has detailed a framework for a wearable, open-source neuroprosthetic system designed to improve hand function in individuals with neurological impairments, particularly those with spinal cord injuries and stroke. By leveraging these principles, this project aims to make advanced assistive technology more accessible and affordable and foster continuous innovation and adaptation, meeting diverse user needs.

The system utilizes digital fabrication methods to enable low-cost prototyping of advanced yet fully customizable components. Android apps and progressive web technology provide a graphical interface accessible through widely available wearable devices, such as smartphones, potentially facilitating telerehabilitation. As an ongoing project, further enhancements in robustness, signal processing, and user interface design are anticipated to refine the system’s effectiveness. Moreover, the integration of flexible electronic materials or novel conductive media, such as ion gels, could potentially enhance the signal stability of dry electrodes.

Future research should focus on encouraging user participation and prototyping of system components, capitalizing on the opportunities for customization that open-source development allows. Adhering to medical device safety standards will be crucial for enabling the use of this system for rehabilitative purposes in real-world settings. Ultimately, this project holds the potential to make a significant impact on the field of assistive technology, providing valuable tools for rehabilitation professionals and individuals with disabilities.

## Data Availability

The device design and software are available in repositories under the MIT License, allowing unrestricted use, modification, and distribution with proper attribution. The repositories and additional resources can be accessed at the following links: [LM_FW] https://github.com/ThorsenRune/LM_FW (accessed on 20 May 2025); [LM_APP] https://github.com/ThorsenRune/LM-Android (accessed on 20 May 2025); [LM_HW] https://github.com/ThorsenRune/LM-HW (accessed on 20 May 2025); [LM_ElAp] https://github.com/ThorsenRune/LM_ElAp (accessed on 20 May 2025); [Wiki] https://github.com/ThorsenRune/MeCFES/wiki (accessed on 20 May 2025); [Video] https://www.youtube.com/watch?v=vWHs3x3pt_E (accessed on 20 May 2025); [Solvespace] https://github.com/ThorsenRune/solvespace (accessed on 20 May 2025).

## Abbreviations

The following abbreviations are used in this manuscript:

CSCI	Cervical spinal cord injury
FES	Functional electrical stimulation
MES	Myoelectric signal
PCB	Printed circuit board
